# Supplementary material for: Direct-Acting Antivirals for HCV Treatment in Decompensated Liver Cirrhosis Patients: A Systematic Review and Meta-Analysis
Source: J Pers Med. 2022 Sep 15;12(9):1517. doi: 10.3390/jpm12091517 (PMC9506163; doi:10.3390/jpm12091517)
Supplement: Supplementary file 1 [file jpm-12-01517-s001.zip › jpm-1875576-supplementary.pdf]

Supplementary Material

Direct-acting antivirals for HCV treatment in decompensated liver cirrhosis patients: A systematic review and meta-analysis

Table of Contents

I. Supplementary Materials and Methods……………………….……………….……..2

Search strategy

II. Supplementary Tables and Figures…………………………………………………6

Supplementary figures

Risk of bias

Publication bias

I. Search strategy

1-1. Ovid MEDLINE(R)

No.	Search terms
1	exp Hepatitis C, Chronic/
2	chronic adj5 (hepatitis C or hepacivirus* or hcv).mp.
3	1 OR 2
4	exp Antiviral agents/
5	exp Protease Inhibitors/
6	exp Nucleic acid synthesis inhibitors/
7	(direct* acting anti?viral*) or DAA or ((protease or polymerase) adj3 inhibitor*).mp.
8	(ledipasvir OR sofosbuvir OR elbasvir OR grazoprevir OR velpatasvir OR voxilaprevir OR glecaprevir OR pibrentasvir OR ombitasvir OR paritaprevir OR ritonavir OR dasabuvir OR simeprevir OR boceprevir OR telaprevir OR asunaprevir OR daclatasvir OR Harvoni OR Zepatier OR Epclusa OR Vosevi OR

	Mavyret OR Maviret OR 'Holkira Pak'OR 'Viekira Pak'OR Viekirax OR Exviera OR Technivie OR Galexos OR Sovaldi OR Victrelis OR Incivek OR Incivo OR Olysio OR Sunvepra OR Daklinza).mp.
9	4 or 5 or 6 or 7 or 8
10	3 and 9
11	(Randomized Controlled Trials as Topic/ or randomized controlled trial/ or Random Allocation/ or Double Blind Method/ or Single Blind Method/ or clinical trial/ or clinical trial, phase i.pt. or clinical trial, phase ii.pt. or clinical trial, phase iii.pt. or clinical trial, phase iv.pt. or controlled clinical trial.pt. or randomized controlled trial.pt. or multicenter study.pt. or clinical trial.pt. or exp Clinical Trials as topic/ or (clinical adj trial$).tw. or ((singl$ or doubl$ or treb$ or tripl$) adj (blind$3 or mask$3)).tw. or PLACEBOS/ or placebo$.tw. or randomly allocated.tw. or (allocated adj2 random$).tw.) not (case report.tw. or letter/ or historical article/)
12	Epidemiologic Studies/ or exp Case Control Studies/ or exp Cohort Studies/ or Case-control.tw. or (cohort adj (study or studies)).tw. or Cohort analy$.tw. or (Follow up adj (study or studies)).tw. or (observational adj (study or studies)).tw. or Longitudinal.tw. or Retrospective.tw. or Cross sectional.tw. or Cross-sectional studies/
13	11 OR 12
14	10 AND 13
15	animals/ not (human/ and animals/)
16	14 NOT 15
17	limit 16 to yr="2010 -Current"

1-2. OVID-Embase

No.	Keywords
1	exp Chronic Hepatitis C/
2	chronic adj5 (hepatitis C or hepacivirus* or hcv).mp.
3	1 OR 2
4	exp Antivirus agent/
5	exp Proteinase Inhibitor/

6	exp Nucleic acid synthesis inhibitor/
7	(direct* acting anti?viral*) or DAA or ((protease or polymerase) adj3 inhibitor*).mp.
8	(ledipasvir OR sofosbuvir OR elbasvir OR grazoprevir OR velpatasvir OR voxilaprevir OR glecaprevir OR pibrentasvir OR ombitasvir OR paritaprevir OR ritonavir OR dasabuvir OR simeprevir OR boceprevir OR telaprevir OR asunaprevir OR daclatasvir OR Harvoni OR Zepatier OR Epclusa OR Vosevi OR Mavyret OR Maviret OR "Holkira Pak"OR "Viekira Pak"OR Viekirax OR Exviera OR Technivie OR Galexos OR Sovaldi OR Victrelis OR Incivek OR Incivo OR Olysio OR Sunvepra OR Daklinza).mp.
9	4 or 5 or 6 or 7 or 8
10	3 and 9
11	(Clinical Trial/ OR Randomized Controlled Trial/ OR controlled clinical trial/ OR multicenter study/ OR Phase 3 clinical trial/ OR Phase 4 clinical trial/ OR exp RANDOMIZATION/ OR Single Blind Procedure/ OR Double Blind Procedure/ OR Crossover Procedure/ OR PLACEBO/ OR randomi?ed controlled trial$.tw. OR rct.tw.<br>OR (random$ adj2 allocat$).tw. OR single blind$.tw. OR double blind$.tw. OR ((treble or triple) adj blind$).tw. OR placebo$.tw. OR Prospective Study/) not (Case Study/ OR case report.tw. OR abstract report/ or letter/ OR Conference proceeding.pt. OR Conference abstract.pt. OR Editorial.pt. OR Letter.pt. OR Note.pt.)
12	Clinical study/ OR Case control study OR Family study/ OR Longitudinal study/ OR Retrospective study OR (Prospective study/ not Randomized controlled trials/) OR Cohort analysis/ OR (Cohort adj (study or studies)).mp. OR (Case control adj (study or studies)).tw.OR (follow up adj (study or studies)).tw.OR (observational adj (study or studies)).tw.OR (epidemiologic$ adj (study or studies)).tw. OR (cross sectional adj (study or studies)).tw.
13	11 OR 12
14	10 AND 13
15	animals/ not (human/ and animals/)
16	14 NOT 15
17	limit 16 to yr="2010 -Current"

1-3. Cochrane Register of Controlled Trials (CENTRAL)

No.	Keywords
1	[Hepatitis C, Chronic] explode all trees
2	chronic near/5 (hepatitis C or hepacivirus* or hcv)
3	#1 OR #2
4	MeSH descriptor: [Antiviral Agents] explode all trees
5	MeSH descriptor: [Protease Inhibitors] explode all trees
6	MeSH descriptor: [Nucleic Acid Synthesis Inhibitors] explode all trees
7	(direct* acting anti?viral*) or DAA or ((protease or polymerase) near/3 inhibitor*)
8	(ledipasvir OR sofosbuvir OR elbasvir OR grazoprevir OR velpatasvir OR voxilaprevir OR glecaprevir OR pibrentasvir OR ombitasvir OR paritaprevir OR ritonavir OR dasabuvir OR simeprevir OR boceprevir OR telaprevir OR asunaprevir OR daclatasvir OR Harvoni OR Zepatier OR Epclusa OR Vosevi OR Mavyret OR Maviret OR 'Holkira Pak'OR 'Viekira Pak'OR Viekirax OR Exviera OR Technivie OR Galexos OR Sovaldi OR Victrelis OR Incivek OR Incivo OR Olysio OR Sunvepra OR Daklinza)
9	#4 or #5 or #6 or #7 or #8
10	#3 and #9
11	#10 with Publication Year from 2010 to 2020, in Trials

1-4 Local databases

Local Database	Search strategy
Kmbase	("hepatitis C" OR hepacivirus OR HCV) AND ("direct acting antiviral" OR DAA)
	("hepatitis C" OR hepacivirus OR HCV) AND (ledipasvir OR sofosbuvir OR elbasvir OR grazoprevir OR velpatasvir OR voxilaprevir OR glecaprevir OR pibrentasvir OR ombitasvir OR paritaprevir OR ritonavir OR dasabuvir OR simeprevir OR boceprevir OR telaprevir OR asunaprevir OR daclatasvir OR Harvoni OR Zepatier OR Epclusa OR Vosevi OR Mavyret OR Maviret OR "Holkira Pak"OR "Viekira Pak"OR Viekirax OR Exviera OR Technivie OR Galexos OR Sovaldi OR Victrelis OR Incivek OR Incivo OR Olysio OR Sunvepra OR Daklinza)

	Overall results after excluding duplicates
Kmbase	(“hepatitis C” OR hepacivirus OR HCV) AND “direct acting antiviral“
	(“hepatitis C” OR hepacivirus OR HCV) AND DAA
	(ledipasvir OR sofosbuvir OR elbasvir OR grazoprevir OR velpatasvir OR voxilaprevir OR glecaprevir OR pibrentasvir OR ombitasvir OR paritaprevir OR ritonavir OR dasabuvir OR simeprevir OR boceprevir OR telaprevir OR asunaprevir OR daclatasvir OR Harvoni OR Zepatier OR Epclusa OR Vosevi OR Mavyret OR Maviret OR “Holkira Pak”OR “Viekira Pak”OR Viekirax OR Exviera OR Technivie OR Galexos OR Sovaldi OR Victrelis OR Incivek OR Incivo OR Olysio OR Sunvepra OR Daklinza)
	(([ALL=C형 간염] OR [ALL=C형간염]) AND ([ALL=항바이러스제] OR [ALL=항 바이러스제]))
	Overall results after excluding duplicates
RISS	(“hepatitis C” OR hepacivirus OR HCV) AND “direct acting antiviral"
	(“hepatitis C” OR hepacivirus OR HCV) AND DAA
	(ledipasvir OR sofosbuvir OR elbasvir OR grazoprevir OR velpatasvir OR voxilaprevir OR glecaprevir OR pibrentasvir OR ombitasvir OR paritaprevir OR ritonavir OR dasabuvir OR simeprevir OR boceprevir OR telaprevir OR asunaprevir OR daclatasvir OR Harvoni OR Zepatier OR Epclusa OR Vosevi OR Mavyret OR Maviret OR “Holkira Pak”OR “Viekira Pak”OR Viekirax OR Exviera OR Technivie OR Galexos OR Sovaldi OR Victrelis OR Incivek OR Incivo OR Olysio OR Sunvepra OR Daklinza)
	C형 간염 AND 항 바이러스제
	C형간염 AND 항바이러스제
	Overall results after excluding duplicates

II. Supplementary figures

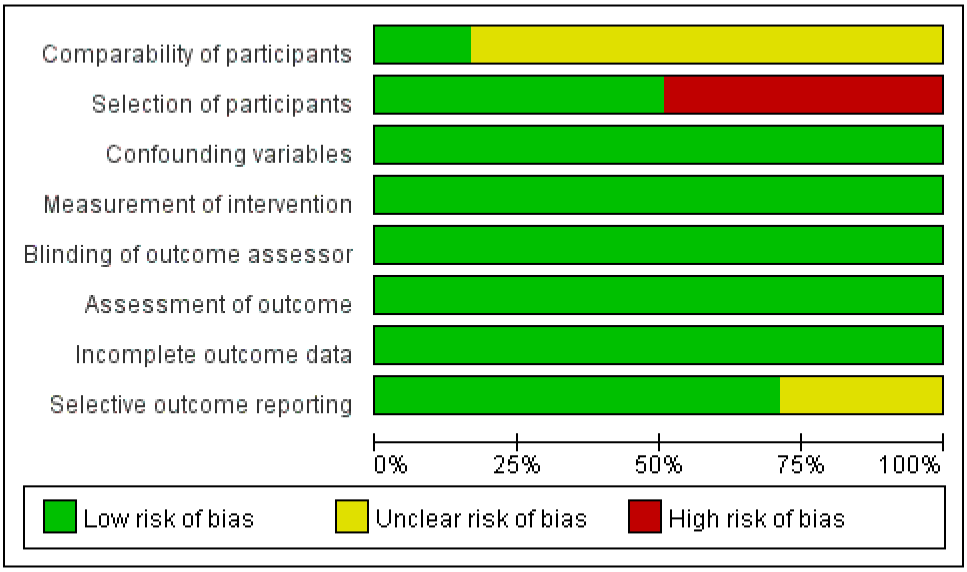
Comparability of participants
Selection of participants
Confounding variables
Measurement of intervention
Blinding of outcome assessor
Assessment of outcome
Incomplete outcome data
Selective outcome reporting
0%
25%
50%
75%
100%
Low risk of bias
Unclear risk of bias
High risk of bias

	Comparability of participants	Selection of participants	Confounding variables	Measurement of intervention	Blinding of outcome assessor	Assessment of outcome	Incomplete outcome data	Selective outcome reporting
Afdhal 2017	?	+	+	+	+	+	+	+
Alonso 2017	?	−	+	+	+	+	+	?
Backus 2016	+	−	+	+	+	+	+	?
Belli 2016	?	−	+	+	+	+	+	+
Belli 2018	?	−	+	+	+	+	+	+
Berkan- Kawińska 2021	+	−	+	+	+	+	+	+
Bittermann 2021	?	−	+	+	+	+	+	?
Calleja, 2017	?	−	+	+	+	+	+	?
Calvaruso 2018	?	+	+	+	+	+	+	+
Carrillo 2017	?	−	+	+	+	+	+	+
Charlton 2015	?	+	+	+	+	+	+	+
Chatterjee 2019	?	−	+	+	+	+	+	+
Cheung 2016	?	+	+	+	+	+	+	+
Curry 2015	?	+	+	+	+	+	+	+
Curry 2016	?	+	+	+	+	+	+	+
Debnath 2019	?	−	+	+	+	+	+	?
Deterding 2015	?	+	+	+	+	+	+	?
Essa 2019	?	+	+	+	+	+	+	+
Flemming 2017	?	−	+	+	+	+	+	+
Foster 2016	?	+	+	+	+	+	+	+
Garg 2018	?	+	+	+	+	+	+	+
Gentile 2019	?	+	+	+	+	+	+	+
Goel 2017	+	+	+	+	+	+	+	+
Gupta 2018	?	+	+	+	+	+	+	+
Hanafy 2019	?	−	+	+	+	+	+	+
Ibrahim 2021	+	−	+	+	+	+	+	?
Jacobson 2019	+	+	+	+	+	+	+	+
Krassenburg 2020	+	−	+	+	+	+	+	+
Kumada 2021	?	+	+	+	+	+	+	+
Kwo 2018	?	+	+	+	+	+	+	+
Lens 2017	?	−	+	+	+	+	+	?
Lens 2020	?	+	+	+	+	+	+	+
Lim 2018	?	+	+	+	+	+	+	?
Lobato 2019	?	+	+	+	+	+	+	?
Lourenco 2021	?	−	+	+	+	+	+	+
Maan 2016	?	−	+	+	+	+	+	+
Macken 2019	?	+	+	+	+	+	+	?
Mandorfer 2016	?	−	+	+	+	+	+	?
Mangia 2018	?	+	+	+	+	+	+	+
Manolakopoulos 2017	?	−	+	+	+	+	+	+
Mazzarelli 2018	?	−	+	+	+	+	+	+
McCaughan 2017	?	+	+	+	+	+	+	+
Modi 2016	?	+	+	+	+	+	+	+
Omar El-Sherif 2018	?	−	+	+	+	+	+	+
Papatheodoridis 2017	?	−	+	+	+	+	+	?
Pascasio 2017	?	−	+	+	+	+	+	+
Poordad 2016	+	+	+	+	+	+	+	+
Ridruejo 2019	?	+	+	+	+	+	+	?
Sanai 2018	+	+	+	+	+	+	+	+
Sandmann 2018	?	−	+	+	+	+	+	+
Saxena 2015	+	−	+	+	+	+	+	+
Shiffman 2015	?	−	+	+	+	+	+	?
Steinebrunner 2018	?	−	+	+	+	+	+	?
Tahata 2021	?	+	+	+	+	+	+	+
Terrault 2016	?	+	+	+	+	+	+	?
Tmu 2019	+	−	+	+	+	+	+	+
Verna 2020	?	+	+	+	+	+	+	+
Wei 2020	?	+	+	+	+	+	+	+
Welzel 2016	?	−	+	+	+	+	+	+

Supplementary Figure S1. Risk of bias.

(A)

Study or Subgroup	Decompensated LC Events	Decompensated LC Total	Compensated LC Events	Compensated LC Total	Weight	Odds Ratio M-H, Random, 95% CI
Afdhal 2017	19	28	14	78	3.1%	9.65 [3.62, 25.75]
Alonso 2017	38	42	157	166	2.4%	0.54 [0.16, 1.86]
Backus 2016	173	209	5753	6333	5.6%	0.48 [0.33, 0.70]
Berkan- Kawińska 2021	261	281	2181	2284	5.1%	0.62 [0.38, 1.01]
Calleja, 2017	126	136	2275	2362	4.3%	0.48 [0.24, 0.95]
Calvaruso 2018	189	214	1937	2035	5.2%	0.38 [0.24, 0.61]
Carrillo 2017	112	144	424	451	4.8%	0.22 [0.13, 0.39]
Chatterjee 2019	24	29	21	21	0.6%	0.10 [0.01, 1.98]
Garg 2018	30	41	33	37	2.3%	0.33 [0.10, 1.15]
Goel 2017	23	30	47	51	2.2%	0.28 [0.07, 1.05]
Gupta 2018	30	31	115	120	1.0%	1.30 [0.15, 11.59]
Ibrahim 2021	283	326	271	275	2.9%	0.10 [0.03, 0.27]
Jacobson 2019	27	30	10	10	0.6%	0.37 [0.02, 7.88]
Krassenburg 2020	120	149	647	719	5.2%	0.46 [0.29, 0.74]
Lens 2017	140	176	693	746	5.3%	0.30 [0.19, 0.47]
Lim 2018	213	231	346	358	4.0%	0.41 [0.19, 0.87]
Lobato 2019	50	66	740	809	4.6%	0.29 [0.16, 0.54]
Maan 2016	83	101	261	304	4.6%	0.76 [0.42, 1.39]
Papatheodoridis 2017	40	53	278	317	4.1%	0.43 [0.21, 0.88]
Pascasio 2017	142	171	62	67	3.1%	0.39 [0.15, 1.07]
Poordad 2016	39	48	11	12	1.0%	0.39 [0.04, 3.46]
Ridruejo 2019	82	91	466	486	3.7%	0.39 [0.17, 0.89]
Sanai 2018	43	48	126	135	2.6%	0.61 [0.20, 1.93]
Saxena 2015	37	49	91	100	3.2%	0.30 [0.12, 0.78]
Shiffman 2015	28	39	70	80	3.2%	0.36 [0.14, 0.95]
Tahata 2021	74	82	100	108	3.0%	0.74 [0.27, 2.06]
Terrault 2016	263	293	1456	1495	5.1%	0.23 [0.14, 0.38]
Tmu N 2019	27	34	24	28	2.1%	0.64 [0.17, 2.47]
Wei 2020	25	26	29	31	0.8%	1.72 [0.15, 20.17]
Welzel 2016	131	165	200	213	4.3%	0.25 [0.13, 0.49]
**Total (95% CI)**		**3363**		**20231**	**100.0%**	**0.43 [0.34, 0.54]**
Total events	2872		18838			

Heterogeneity: Tau² = 0.23; Chi² = 76.32, df = 29 (P < 0.00001); I² = 62%

Test for overall effect: Z = 6.96 (P < 0.00001)

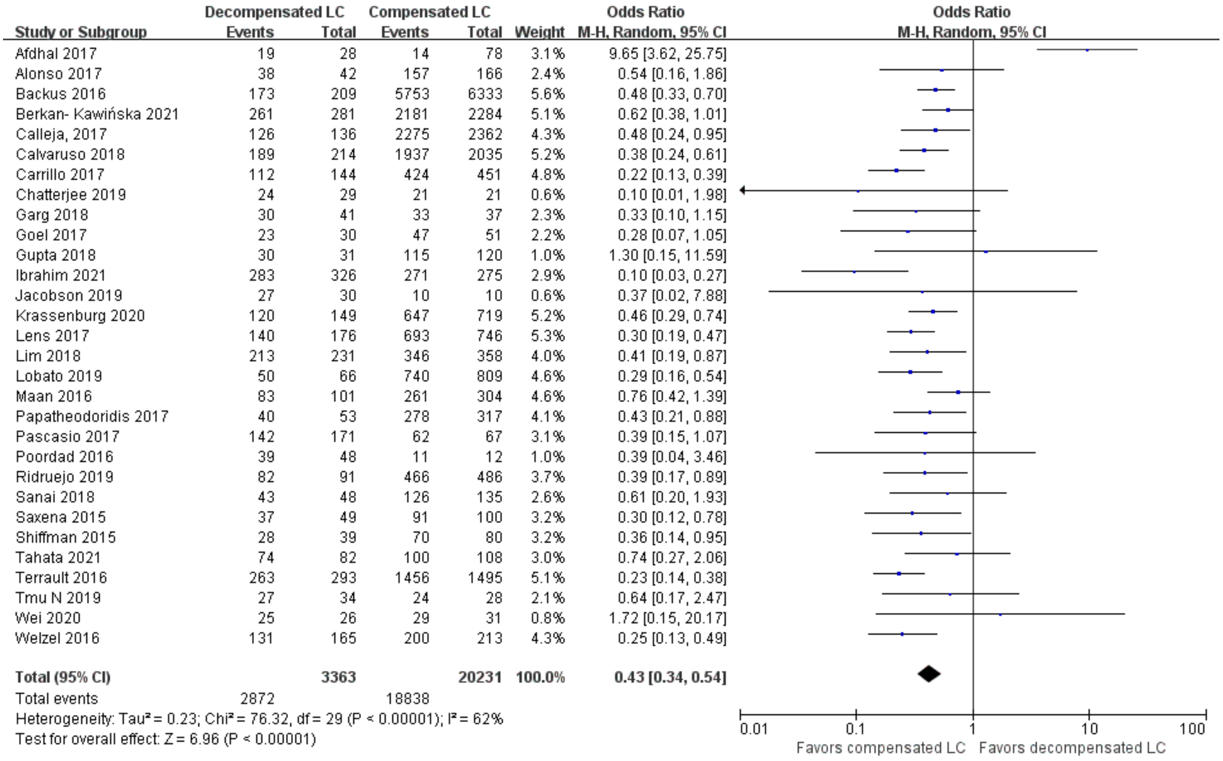

(B)

Study or Subgroup	Decompensated LC Events	Decompensated LC Total	Compensated LC Events	Compensated LC Total	Weight	Odds Ratio M-H, Random, 95% CI	Year
Alonso 2017	2	77	2	131	3.0%	1.72 [0.24, 12.46]	2016
Carrillo 2017	9	175	12	564	13.3%	2.49 [1.03, 6.02]	2017
Sanai 2018	1	48	2	135	2.0%	1.41 [0.13, 15.97]	2018
Mangia 2018	21	250	34	1314	26.6%	3.45 [1.97, 6.06]	2018
Calvaruso 2018	17	214	43	2035	25.4%	4.00 [2.24, 7.14]	2018
Krassenburg 2020	22	149	67	719	29.6%	1.69 [1.00, 2.83]	2020
**Total (95% CI)**		**913**		**4898**	**100.0%**	**2.67 [1.88, 3.79]**	
Total events	72		160				

Heterogeneity: Tau² = 0.04; Chi² = 6.25, df = 5 (P = 0.28); I² = 20%

Test for overall effect: Z = 5.49 (P < 0.00001)

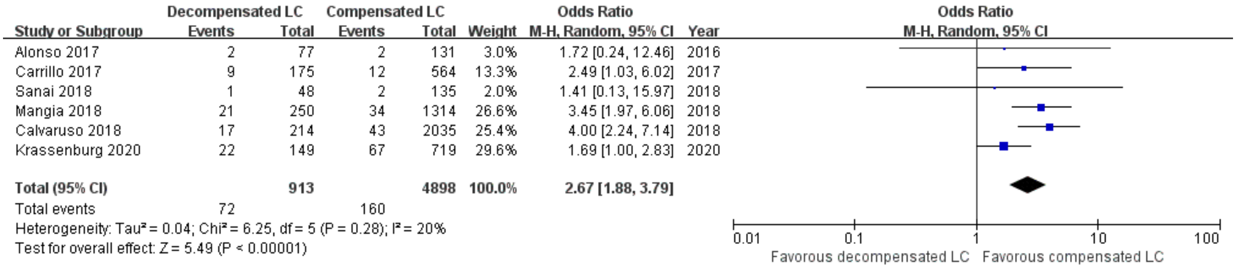

(C)

Study or Subgroup	Decompensated LC Events	Decompensated LC Total	Cecompensated LC Events	Cecompensated LC Total	Weight	Odds Ratio M-H, Random, 95% CI
Alonso 2017	2	77	1	166	2.4%	4.40 [0.39, 49.28]
Berkan- Kawińska 2021	17	316	21	2397	32.4%	6.43 [3.36, 12.33]
Carrillo 2017	11	175	5	564	12.0%	7.50 [2.57, 21.89]
Goel 2017	5	30	2	51	4.7%	4.90 [0.89, 27.07]
Krassenburg 2020	19	149	12	719	24.6%	8.61 [4.08, 18.17]
Maan 2016	5	114	1	319	2.9%	14.59 [1.69, 126.24]
Sanai 2018	2	48	1	165	2.3%	7.13 [0.63, 80.40]
Saxena 2015	1	55	1	101	1.8%	1.85 [0.11, 30.20]
Tahata 2021	2	82	1	108	2.3%	2.67 [0.24, 30.02]
Terrault 2016	7	414	6	917	11.4%	2.61 [0.87, 7.82]
Tmu 2019	10	47	1	28	3.1%	7.30 [0.88, 60.47]
**Total (95% CI)**		**1507**		**5535**	**100.0%**	**6.14 [4.24, 8.89]**
Total events	81		52			

Heterogeneity: Tau² = 0.00; Chi² = 5.25, df = 10 (P = 0.87); I² = 0%

Test for overall effect: Z = 9.60 (P < 0.00001)

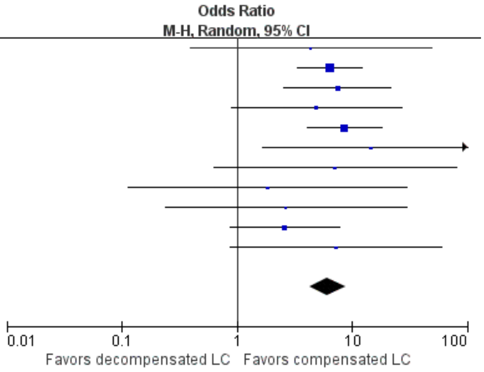

Supplementary Figure S2. Estimated event rate of (A) sustained virologic response of

direct-acting antiviral (DAAs), (B) development of hepatocellular carcinoma, and (C) mortality in decompensated vs. compensated liver cirrhosis patients with chronic hepatitis C virus (HCV) infection. CI, confidence interval.

(A)

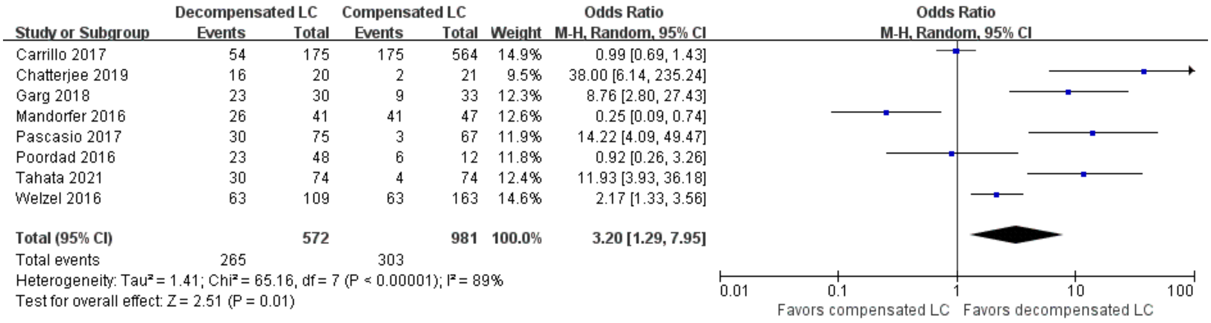

(B)

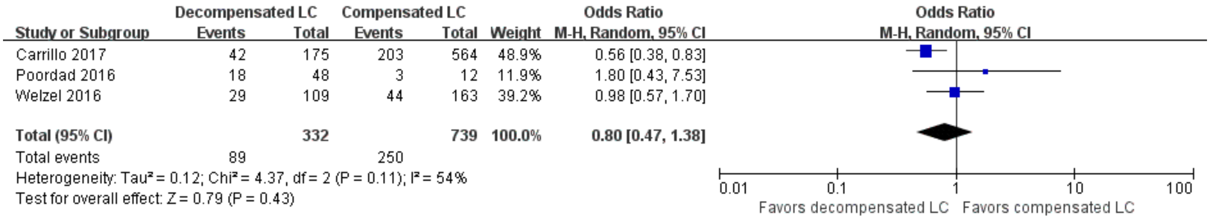

Supplementary Figure S3. Estimated event rate of (A) improvement in liver function and (B) worsening in liver function after direct-acting antiviral (DAA) treatment in decompensated vs. compensated liver cirrhosis patients.
